# Notes and News

**Published:** 1891-11-28

**Authors:** 


### NOTES AND NEWS.

COLLECTED AND COMMUNICATED.

THE late Mr. R. A. Newbon, of the firm of Newbon and Harding, has left the noble legacy of fifteen thousand pounds to the Great Northern Central Hospital. Mr. Newbon had long resided and carried on business in the North of London, in which district the hospital is situated.

THE Dublin Hospital Sunday collection brings to our notice various facts relating to the sick relief institutions of that city. The fund subscribes about 10 per cent. of the required sum for their maintenance. The combined expenditure of all the hospitals amounts to £43 000. No very large sum, considering that 1,200 beds are provided for, and 19,000 out-patients treated, exclusive of those attending at the dispensaries. The official annual inspection of the Dublin hospitals reported favourably of nearly all the institutions, and we are not surprised to learn that the fund has steadily been increasing since its foundation in 1874.

THE Council of the Metropolitan Hospital Sunday Fund have now issued their report for the present year, and the facts before us are not entirely encouraging in respect to the congregational collection of the fund. The metropolitan collection, this year falls short by upwards of £2,000 the amount realised from this source in 1890. That the fund shows a total increase by £2,515 over the amount received in 1890 is due to legacies and special donations being larger than any previous year, the Duke of Cleveland contributing £5,000 and Sir Savil Crosseley £1,000. We congratulate the Fund on such munificent gifts, but a steady reliable return from the congregations of our churches should be striven after rather than dependence being placed on windfalls, which can only occasionally cover deficiences from other sources. There are two other points of note in the report. A provincial institution is quoted as receiving an award in consideration of its providing for London patients. It is stated that provincial communities whilst continually sending large numbers of patients to the London hospitals, refuse to contribute in any manner to the funds of those institutions. This appears to be a very unequal arrangement. Lastly, we see with surprise that four institutions sent in their statistics too late for consideration. After all the fund is to be congratulated that in 1891 their collection from all sources amounted to no less sum than £45,331. If the rate of increase is steady, as we hope it may be, 1893 may see a collection actually double the amount of the first collection in 1873 which will be a period of twenty years before.

WITH the object of endowing a cot in the Victoria Hospital for Children, Chelsea, the members, associates, and graduates of "The Children's Salon" have taken Prince's Hall, Piccadilly, for the 13th and 14th January, when they will be "At Home" to their friends. Only children will take part in the entertainment, particulars of which may be had of Captain Blount, the Secretary, at the hospital.

DR. BOWLAN, who has served for upwards of two and a half years as house surgeon in the Newcastle Union Hospital, has been appointed to a similar office in the St. George's-in-the-East Union Infirmary, London. During his service at Newcastle his gentle manners and skilful treatment made him a general favourite, and the officers of the union, to the number of about thirty, met in the billiard room (kindly granted for the occasion by the guardians), on Wednesday evening, to express their great sorrow at his departure, and their good wish for his future. Dr. Dodd was moved to the chair by Mr. Wilson, who, in a touching address, expressed his high opinion of Dr. Bowlan as a medical man, and the pleasant relationship which existed between them. The Rev. Thomas Averell was called upon to present Dr. Bowlan with the officers' gift, a handsome dressing-case, and in addition to the officers' contribution to the testimonial fund, a large number of the hospital patients requested to be allowed to give their pence to the same object. Probably a more genuine token of respect and good feeling was never presented to a public official. The Rev. I. W. Milner and Mr. William Woodger, guardians, also attended the interesting function, and in very laudatory terms Mr. Milner expressed the universal opinion regarding Dr. Bowlan. After the usual compliments the meeting terminated.

WE have heard a great deal of exaggeration on the subject of hospital scandals of late, and when Londoners take to grumbling they know how to pile up the agony, particularly if the holiday season big gooseberry crop of Fleet Street be somewhat scanty. People gain so erroneous an idea of the average condition of hospitals generally that some mean writers make it a pretext for arguing that the working man's annual Saturday subscription is a mistake, whilst niggards of larger income find in it an excuse for closing their own pockets. The truth, at the worst, is that, to correct the tendency of patients to verbosity, hospital doctors are given to curtness; and having perhaps two minutes to decide on a case where a private practitioner would take half-an-hour, they may now and then make a wrong diagnosis, especially in these days of shams and the inaccuracy of descriptive information. As a rule, however, despite the stress of members and difficult circumstances, they do good work in a practical fashion, and some of the institutions are as near perfection as anything human can be. Between the Eagle and Moorgate Street, for example, is an old City establishment under regulations, management, and staff such as silence even the most cantankerous Cockney critics. The poor married women, for whom it is specially supported, received as tender care and skilful attention as royalty may command for itself. Well might one such patient say that, by contrast with the wear and tear of her own poor home, "It was like a fortnight of Heaven to rest in such an abode of spotless purity, warm comfort, generous diet, and the constant kindness of perfect ladies." There is a good deal of pity lavished and much money wasted in these days on the "residuum" and criminals who, humanly speaking, are moss irredeemable. Philanthropists, who wish rather to help the deserving poor who, by heroic endeavours, manage to keep themselves unsubmerged, cannot find a better way to aid, without pauperising, than by copying for other localities the City of London Lying-in Hospital.—From the *Sheffield Daily Telegraph*, November 5th, 1891.

